# Second-harmonic generation in NbOI_2_-integrated silicon nitride microdisk resonators

**DOI:** 10.1515/nanoph-2025-0428

**Published:** 2025-11-07

**Authors:** Ning Liu, Qiang Liu, Yutian Lin, Zhihong Zhu, Ken Liu

**Affiliations:** College of Advanced Interdisciplinary Studies & Hunan Provincial Key Laboratory of Novel Nanooptoelectronic Information Materials and Devices and Nanhu Laser Laboratory, National University of Defense Technology, Changsha 410073, China

**Keywords:** second-harmonic generation, niobium oxide diiodides, silicon nitride, microdisks, resonators

## Abstract

In recent years, two-dimensional (2D) niobium oxide dihalides (e.g., NbOI_2_) have garnered significant research interest in nonlinear photonics due to their prominent second-order nonlinear optical properties. Integrating these materials with high-quality-factor optical microcavities represents a crucial approach for developing high-performance on-chip nonlinear optical devices. This work demonstrates NbOI_2_-integrated silicon nitride (Si_3_N_4_) microdisk resonators that achieve second-harmonic generation under low-power (sub-milliwatt) continuous-wave laser pumping, leveraging the superior second-order nonlinearity of NbOI_2_ and the strong optical field confinement capability of Si_3_N_4_ microdisks. The conversion efficiency of the device is calculated to be about 0.024 %/W. The intrinsic lack of inversion symmetry in NbOI_2_ crystals avoids the laborious layer-number-dependent symmetry screening typically required for other 2D materials, while the developed van der Waals transfer technique provides a universal strategy for integrating niobium oxide dihalides with photonic microcavities. This study not only establishes a material-photon co-design strategy for on-chip nonlinear light sources but also lays a critical foundation for advancing quantum photonic chips and on-chip metrology systems.

## 1 Introduction

As one of the central processes in second-order nonlinear optical effects, second-harmonic generation (SHG) occupies a critical position in integrated photonics, with its applications extensively permeating cutting-edge fields such as materials science [1], [2], [3], biomedicine [4], [5], and laser technology [6], [7], [8]. High-quality-factor (*Q* factor) whispering gallery mode (WGM) optical microcavities [9], [10], featuring strong optical field confinement capability, significantly enhance light–matter interaction and serve as an ideal platform for the study of integrated nonlinear optics. However, for the widely utilized silicon nitride (Si_3_N_4_) photonic platform, the intrinsic centrosymmetry of the material fundamentally prohibits direct SHG implementation in Si_3_N_4_ microdisk resonators. SHG has been demonstrated in symmetric materials through methods such as interfacial effects [11], [12] or optical-induced nonlinearity [13], [14], [15]. However, achieving efficient SHG under moderate pump power remains a key bottleneck, which limits the expansion of nonlinear functionalities in Si_3_N_4_ microcavity platforms.

Two-dimensional (2D) niobium oxide dihalides (NbOX_2_, X = Cl, Br, I) [16], [17] offer novel opportunities for integrated nonlinear photonics due to their unique layered structure and physical properties. First, the intrinsic lack of inversion symmetry in NbOX_2_ avoids a demanding choice of the layer parity of the 2D materials [18], [19]; Second, via van der Waals (vdW) forces, NbOX_2_ enables universal integration with silicon photonic devices (e.g., Si_3_N_4_ microcavities) [20], [21], effectively circumventing the lattice mismatch constraints inherent to conventional integration processes. While current studies have confirmed the exceptional SHG performance of NbOX_2_ in free-space optical configurations [22], [23], [24], its integration with on-chip optical systems remains unexplored – advancing this direction could propel multifunctional applications of NbOX_2_ in chip-scale nonlinear optical devices.

In this work, SHG was realized in a fabricated NbOI_2_-integrated Si_3_N_4_ microdisk resonator under the pump of a sub-milliwatt continuous-wave (CW) laser and the conversion efficiency was estimated to be 0.024 %/W. The technological breakthrough stems from the following synergistic design strategies: (1) Precise transfer of few-layer NbOI_2_ onto the edge of the Si_3_N_4_ microdisk via a site-specific vdW transfer technique, ensuring interaction between the second-order nonlinear medium and the cavity modes’ evanescent field; (2) Overcoming the optical path length limitation imposed by the nanometer-scale thickness of NbOI_2_ through the resonance of WGMs. Beyond validating the feasibility of 2D NbOX_2_ materials for integrated nonlinear photonics, this work establishes a universal strategy for developing sub-milliwatt threshold on-chip nonlinear light source via a co-design strategy that synergizes “material nonlinearity with cavity field enhancement.”

## 2 Results and discussion

### 2.1 Optical characterization and environmental stability of 2D NbOI_2_

**Figure 1:**
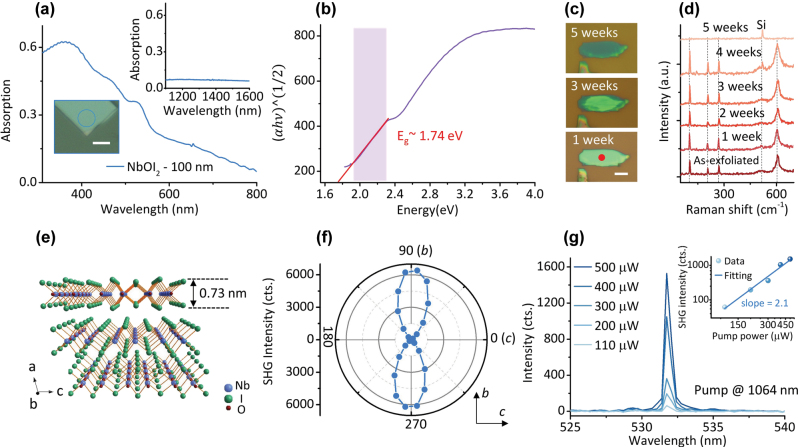
Optical characterization and environmental stability of 2D NbOI_2_. (a) UV-NIR absorption spectra of a NbOI_2_ flake (thickness: 100 nm). The main panel shows absorption in the 300–800 nm range. Top inset: absorption in the telecommunication band (1,100–1,600 nm); bottom inset: optical microscopy (OM) image of the measured sample (blue circle marks the test region; scale bar: 5 μm). (b) Curve of the ${\left(\alpha h\nu \right)}^{1/2}-h\nu$ relationship based on the Tauc plot method (purple shaded area indicates the linear fitting region), determining a bandgap of 1.74 eV for the 100 nm-thick NbOI_2_. (c) OM images of time-dependent degradation of a NbOI_2_ flake (initial thickness 30 nm) under ambient conditions (scale bar: 5 μm). Red dot in the bottom panel marks the Raman test position for Figure 1d. (d) Evolution of Raman spectra for the sample in (c): initial state displays characteristic NbOI_2_ peaks (104 cm^−1^, 208 cm^−1^, 271 cm^−1^, 608 cm^−1^); after five weeks, only the Si substrate peak at 521 cm^−1^ remains (gray dashed lines indicate the peak positions). (e) 3D schematic of the NbOI_2_ crystal structure: the *a*-axis corresponds to the vdW stacking direction with a monolayer thickness of 0.73 nm. (f) Polarization-resolved SHG polar plot: second-harmonic signal intensity exhibits a two-lobed distribution, with maximum response along the *b*-axis (θ = 90°). (g) Pump power-dependent SHG spectra of a NbOI_2_ flake under the pump of a picosecond pulsed laser at 1,064 nm. Inset: log plot of the SHG intensity versus pump power, showing a linear fit slope of 2.1, confirming that it is a second-order nonlinear optical process.

Layered NbOI_2_ nanosheets were prepared from NbOI_2_ single crystals using the conventional mechanical exfoliation method (Figure S1). Ultraviolet (UV) – near-infrared (NIR) absorption spectra (Figure 1a) reveals strong absorption for a 100 nm-thick NbOI_2_ flake in the visible range (400–700 nm), while the absolute absorption at the targeted pump wavelength band (around 1,550 nm, inset of Figure 1a) and the second harmonic region (near 775 nm) is below 10 %, indicating the material’s suitability for subsequent SHG studies. The bandgap values of the 100 nm- and 80 nm-thick NbOI_2_ were calculated as 1.74 eV and 1.92 eV, respectively, based on Tauc plot analysis method (Figures 1b and S2) by plotting (*αhν*)^1/2^ versus *hν* [25]. These results align with literature reports [26], [27]. Under ambient conditions (relative humidity ∼60 %), 30 nm-thick NbOI_2_ on a SiO_2_/Si substrate exhibited progressive edge degradation over time (Figures 1c and S3). After 5 weeks, the material fully degraded, with the characteristic Raman peaks of NbOI_2_ (located at 104 cm^−1^, 208 cm^−1^, 271 cm^−1^, 608 cm^−1^) disappearing, leaving only the Si substrate peak at 521 cm^−1^ (Figure 1d). Correspondingly, the second-order nonlinear optical properties in NbOI_2_ also disappeared. For the degradation of the NbOX_2_ materials, the current general solution strategy is to use a thin layer of hexagonal boron nitride (hBN) for packaging [22], which can significantly improve the stability and power threshold of the materials in air.

The NbOI_2_ crystal belongs to the monoclinic *C*2 space group (Figure 1e) with a monolayer thickness of 0.73 nm. Along the *a*-axis, the layers are bonded via vdW forces, with each NbOI_2_ layer composed of NbOI_2_ octahedra; along the *c*-axis, NbI_4_ chains are interconnected through I atoms, where first-order Peierls distortion induces alternating Nb–Nb bond lengths (3.17 Å ↔ 4.35 Å, Figure S4a); along the *b*-axis, the structure is connected via O atoms, and second-order Peierls distortion causes Nb ions to deviate from the centers of the octahedra (displacement: 0.14 Å, Figure S4b), resulting in strong spontaneous polarization along the *b*-axis. Polarization-resolved SHG measurement revealed a characteristic *C*2 symmetry pattern – a two-lobed pattern (Figure 1f), with maximum intensity observed when the pump polarization aligns with the *b*-axis (*θ* = 90°), consistent with theoretical predictions. Under the pump of a 1,064 nm ps pulsed laser, a prominent SHG signal was detected at 532 nm (Figure 1g). The quadratic dependence of SHG intensity on pump power in a log scale (fitted slope: 2.1, inset in Figure 1g) confirms its intrinsic second-order nonlinear optical process.

### 2.2 Integration of 2D NbOI_2_ and Si_3_N_4_ microdisk

**Figure 2:**
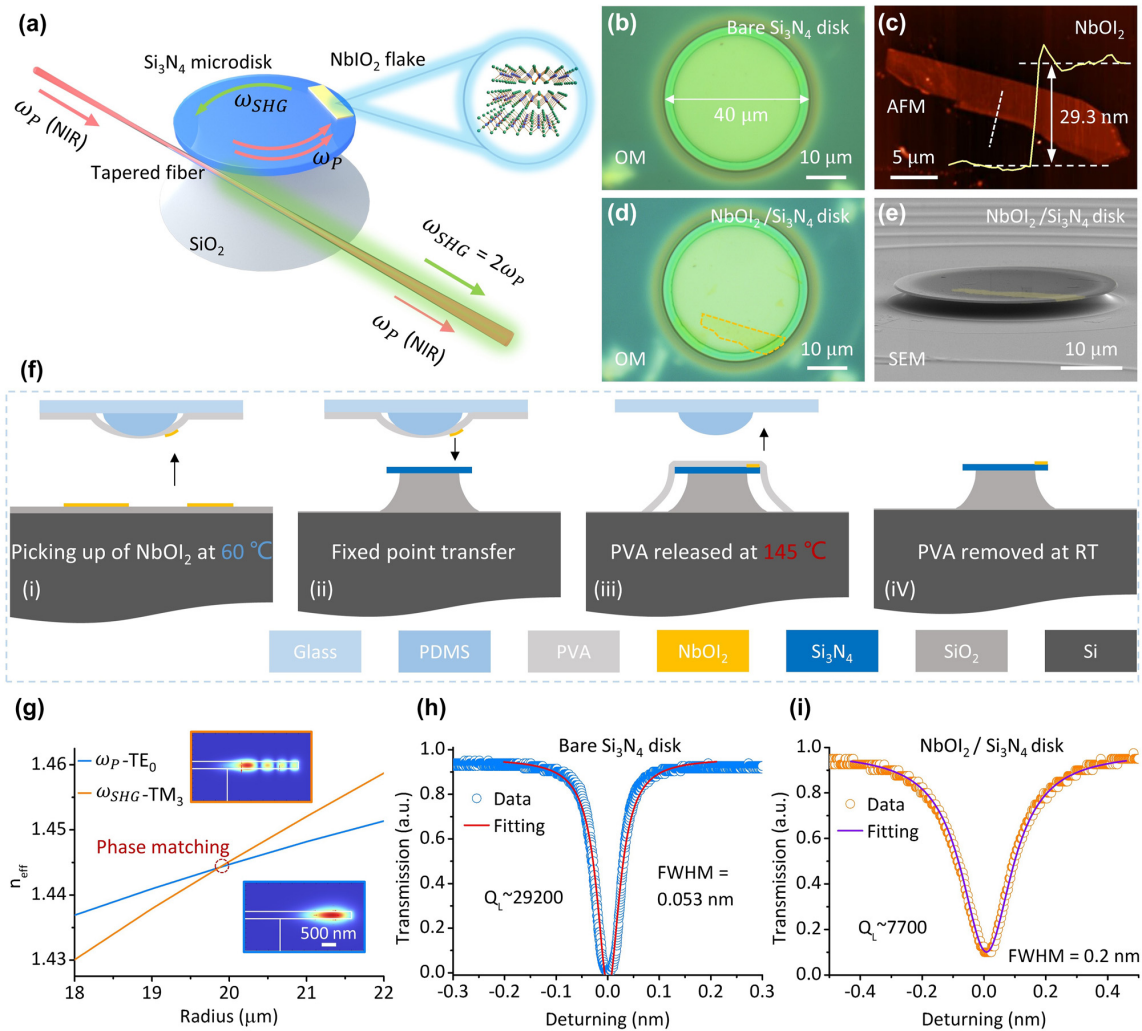
Integration of NbOI_2_ and Si_3_N_4_ microdisk resonator. (a) Schematic of the SHG process in the NbOI_2_-integrated Si_3_N_4_ microdisk resonator: NIR pump light (*ω*_*P*_) is coupled into the Si_3_N_4_ microdisk via a tapered fiber. The interaction between NbOI_2_ flake and the evanescent field of the WGMs generates the second-harmonic signal (*ω*_SHG_), which is coupled out through the same tapered fiber. (b) OM image of a bare Si_3_N_4_ microdisk used in the experiment, with a diameter of 40 μm. (c) Atomic force microscopy (AFM) image of the NbOI_2_ flake used in the experiment. Inset (yellow line): height profile along the white dashed line reveals a thickness of 29.3 nm. (d) OM image of the NbOI_2_-integrated Si_3_N_4_ microdisk resonator, with the NbOI_2_ integration region marked by an orange dashed line. (e) False-colored scanning electron microscopy (SEM) image of the NbOI_2_-integrated Si_3_N_4_ microdisk, where NbOI_2_ is treated in yellow. (f) Flowchart of the multi-step thermally-controlled transfer process: (i) picking up of NbOI_2_ by PDMS-PVA stamp at 60 °C; (ii) transferring the NbOI_2_ to the edge of the Si_3_N_4_ microdisk; (iii) thermally pressurizing and releasing the PVA-NbOI_2_ at 145 °C; (iV) removing PVA in deionized water and completing the integration. (g) Effective refractive index (*n*_eff_) curves of the TE_0_ mode at the fundamental frequency (*ω*_*P*_-TE_0_, blue line) and the TM_3_ mode at the second harmonic frequency (*ω*_SHG_-TM_3_, orange line) as functions of the microdisk diameter. The intersection at a radius of ∼20 μm indicates the phase-matching point. Insets: electric field distributions of the TE_0_ (fundamental frequency) and TM_3_ (second-harmonic frequency) modes. (h, i) Transmission spectra at the resonance wavelengths of the Si_3_N_4_ microdisk before (h) and after (i) the integration of NbOI_2_, with the loaded *Q* factor (*Q*_L_) decreases from 29,200 to 7,700. Circles: experimental data; solid lines: Lorentzian fitting curves.

As shown in Figure 2a, second-order nonlinearity can be endowed to the Si_3_N_4_ microdisk by precisely transferring a NbOI_2_ flake to the edge of the suspended Si_3_N_4_ microdisk through the vdW integration technique. When the pump light (*ω*_*P*_, around 1,550 nm) in the NIR band is coupled with the NbOI_2_-integrated microdisk cavity through a tapered fiber, resonance enhancement occurs at specific wavelengths. The evanescent field of the WGMs interacts with the NbOI_2_ flake, driving the SHG process. The generated second-harmonic signal (*ω*_SHG_ = 2*ω*_*P*_) is then coupled out from the NbOI_2_-integrated microcavity via the tapered fiber, achieving the extraction of the nonlinear signal.

Figure 2b shows a Si_3_N_4_ microdisk used in the experiment. The suspended Si_3_N_4_ microdisk was prepared using a hybrid etching process (Figure S5): a 300 nm-thick Si_3_N_4_ film was deposited on a Si/SiO_2_ (3 μm) substrate by plasma-enhanced chemical vapor deposition (PECVD); UV lithography was employed to pattern the photoresist layer, followed by reactive ion etching (RIE) to transfer the microdisk pattern to the Si_3_N_4_ layer; the release of Si_3_N_4_ microdisks was achieved using 80 °C KOH solution (1/3 mol/L) to etch the SiO_2_ layer (etching rate ∼100 nm/h, Figure S6). By precisely controlling the etching duration (29–30 h), a 0–100 nm-thick SiO_2_ sacrificial layer was retained (Figure S7), ensuring structural stability of the microdisk during subsequent transfer of 2D NbOI_2_.

The vdW integration of 2D NbOI_2_ and Si_3_N_4_ microdisk was achieved using the multi-step thermal-controlled transfer method illustrated in Figure 2f: a polydimethylsiloxane (PDMS)-polyvinyl alcohol (PVA) stamp was used to pick up the NbOI_2_ (Figures 2c and S8a) from the SiO_2_/Si substrate at 60 °C (for 1 min); the picked-up NbOI_2_ was then aligned to the microdisk edge using a high-precision nano-manipulation stage; followed by hot-pressing release (for 5 min) of PVA-NbOI_2_ onto the microdisk at 145 °C (Figures 2f(iii) and S8b); finally, the PVA was dissolved in deionized water, yielding the NbOI_2_-integrated Si_3_N_4_ microdisk (Figure 2d and e). After transfer, the NbOI_2_ adhered to the suspended edge of the Si_3_N_4_ microdisk via vdW force. Assuming a monolayer thickness of 0.73 nm for NbOI_2_, the integrated NbOI_2_ on the microdisk measures ∼29.3 nm in thickness (yellow line in Figure 2c), corresponding to approximately 40 layers. As indicated by prior research [23], within a certain thickness range, the intensity of the second-harmonic signal scales quadratically with the number of layers. The layer-independent broken centrosymmetry in NbOI_2_ crystals significantly reduces the screening time of 2D materials for the fabrication of the integrated structure.

Achieving SHG in bulk materials requires precise matching of the wavevectors of the pump and the second-harmonic. However, in integrated optical microcavities, the paradigm for phase matching undergoes a transformation: it shifts toward matching the effective mode refractive indices at the fundamental and second-harmonic frequencies. These two approaches are fundamentally equivalent in their underlying principles. In order to satisfy the phase matching condition [28], [29], we precisely designed the geometric parameters of the Si_3_N_4_ microdisk resonator. In the simulation, the thickness of the Si_3_N_4_ microdisk was set to 300 nm; the refractive index of Si_3_N_4_ was set to be 2 and did not vary with wavelength. The pump wavelength is set to 1,550 nm, with the corresponding second-harmonic wavelength at 775 nm. It can be seen from Figure 2g that the transverse electric fundamental mode at the fundamental frequency (*ω*_*P*_-TE_0_, blue line) and the transverse magnetic third-order mode at the second harmonic frequency (*ω*_SHG_-TM_3_, orange line) have an intersection of the effective refractive index (*n*_eff_) curves at microdisk radius close to 20 μm, i.e., the phase-matching point. At this point, both energy and momentum conservation are satisfied for the SHG process. The bottom and top insets in Figure 2g illustrate the electric field distributions of the TE_0_ mode at the fundamental frequency and the TM_3_ mode at the second-harmonic frequency, respectively, for a 20 μm-radius Si_3_N_4_ microdisk. We have also analyzed the phase-matching condition of the Si_3_N_4_ microdisk integrated with a 30 nm-thick NbOI_2_ flake (Figure S9). Although the NbOI_2_ affects the effective refractive indices of both fundamental and second-harmonic modes, phase matching can still be achieved at a microdisk diameter close to 20 μm. This approach of achieving phase matching by designing the geometry of microcavities is indeed highly versatile and robust, serving as a powerful tool for engineering a wide range of nonlinear processes (Supporting Material Note S1).

Figure 2h and i show the transmission spectra at resonance wavelengths before and after the integration of NbOI_2_ with the Si_3_N_4_ microdisk, respectively, where Lorentzian fittings of the data (circles) yield the *Q* values of the microdisk resonator. After the integration of NbOI_2_, the loaded *Q* value (*Q*_L_) of the microdisk resonator decreased from 29,200 (Figure 2h) to 7,700 (Figure 2i), primarily due to increased scattering losses at the microdisk edge. Meanwhile, the decrease of the *Q* value also indicates that the WGMs in the Si_3_N_4_ microdisk interacted effectively with the integrated NbOI_2_.

Since high *Q* factor is essential for high frequency conversion efficiency, after transferring NbOI_2_ to the Si_3_N_4_ microdisk we grew a certain thickness of Al_2_O_3_ by atomic layer deposition (ALD), trying to improve the *Q* value of the NbOI_2_-integrated Si_3_N_4_ microdisk [30]. However, no obvious increase in *Q* value was found. Future studies should investigate strategies to mitigate the reduction in the microcavity *Q*-factor, including: (1) Optimizing the integration process: adopting cleaner dry transfer techniques (e.g., direct peeling using PDMS/polycarbonate (PC) films) to minimize contaminants/polymers introduced during transfer; optimizing annealing processes to reduce scattering centers as much as possible. (2) Using hBN encapsulation: covering NbOI_2_ with a layer of hBN before integrating it with the Si_3_N_4_ microdisk resonator. hBN not only enhances the stability of NbOI_2_ in ambient conditions but also, due to its atomically flat surface, reduces the strength of interaction between the optical field and defects/edges of NbOI_2_, thereby significantly lowering scattering losses. (3) Optimization of the microdisk design and post-integration cleaning procedures.

### 2.3 SHG in the NbOI_2_-integrated Si_3_N_4_ microdisk resonator

After the integration of NbOI_2_ and Si_3_N_4_ microdisk resonator, the evanescent field of the WGMs in the Si_3_N_4_ microdisk resonator interacts with the NbOI_2_ flake, enabling SHG under the pump of a sub-milliwatt CW laser. As indicated in Figure 1c and d, bare NbOI_2_ gradually degrades in ambient conditions, with a degradation period of approximately five weeks under our laboratory conditions. For our SHG study, all SHG measurements were completed within 1–2 days after device fabrication, ensuring that the optical properties and nonlinear response remained stable during the measurements. Therefore, the reported experimental results were not affected by material degradation. Besides, the challenge of long-term operational stability for the NbOI_2_-integrated device can be effectively addressed through established 2D material encapsulation techniques, such as hBN and polymethyl methacrylate (PMMA) [22].

**Figure 3:**
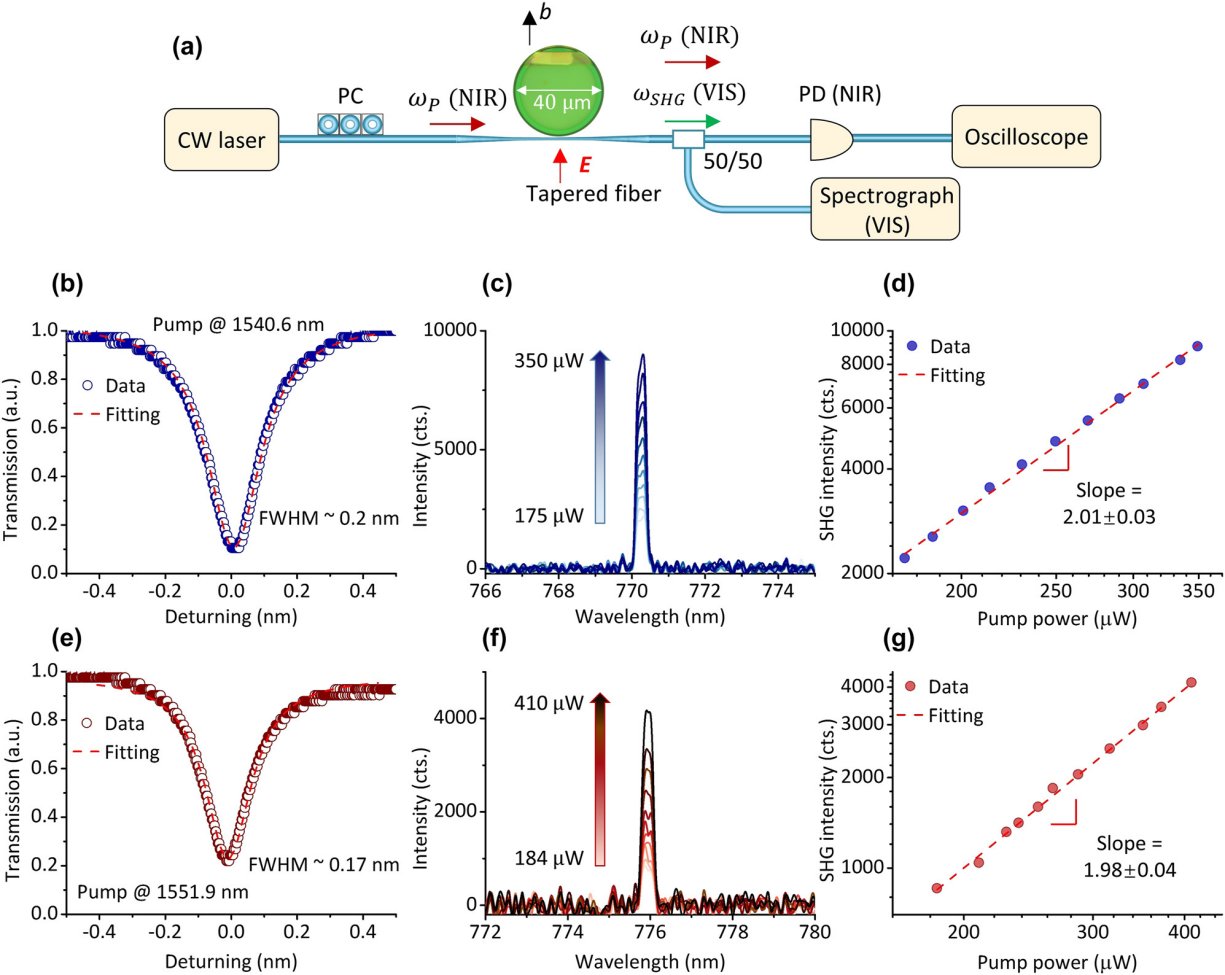
SHG in the NbOI_2_-integrated Si_3_N_4_ microdisk resonator. (a) Schematic of the experimental setup: a tapered fiber is used for the input of the pump (*ω*_*P*_) and the extraction of the second harmonic signal (*ω*_SHG_) from the microdisk; the polarization state of the input light is controlled by a polarization controller (PC), the red arrow indicates the direction of the pump electric field; a photodetector (PD) and oscilloscope record the transmission spectrum of the pump in the NIR band, while a visible (VIS) spectrograph collects the SHG signal. Inset: OM image of the NbOI_2_-integrated Si_3_N_4_ microdisk resonator, the black arrow indicates the direction of the *b*-axis of the integrated NbOI_2_ crystal. (b) Transmission spectrum at the resonant wavelength of 1,540.6 nm, with a Lorentzian-fitted linewidth of 0.2 nm. (c) SHG spectra under varying pump powers. The second harmonic wavelength is locked at 770.3 nm, corresponding to half of the pump wavelength. (d) The relationship between the SHG intensity and the pump power in a log scale. The fitting slope of 2.01 ± 0.03 verifies the intrinsic second-order nonlinear process. (e–g) SHG characterization at a resonant wavelength of 1,551.9 nm. (e) Transmission spectrum with a Lorentzian-fitted linewidth of 0.17 nm. (f) SHG spectra under different pump powers, with the SHG wavelength located at 776 nm, corresponding to half of the pump wavelength. (g) The relationship between the SHG intensity and the pump power in a log scale. The fitting slope of 1.98 ± 0.04 verifies the intrinsic second-order nonlinear process.

Figure 3a illustrates the experimental setup for the measurement, in which the pump light is coupled with the NbOI_2_-integrated Si_3_N_4_ microcavity through a tapered fiber (waist diameter: ∼1 μm), and the polarization state is adjusted by a polarization controller (PC); when the emission wavelength of the tunable laser matches the resonant wavelength of the NbOI_2_-integrated Si_3_N_4_ microcavity, the optical field inside the cavity is resonantly enhanced, driving SHG in the NbOI_2_ flake; the generated second harmonic signal is coupled out through the tapered fiber and detected by a spectrograph for the VIS light; a photodetector (PD, Daheng Optics,DH-GDT-D002N) and an oscilloscope (OWON Technology Inc., VDS1022) are used to record the transmission spectrum for the pump light; the output power for the pump is measured using an optical power meter (Daheng Optics, GCI-080201).

The *b*-axis of the NbOI_2_ crystal (marked with a black arrow) is tangential to the edge of the microdisk, i.e., aligned along the radial direction of the microdisk. In practice, during our experimental testing, by adjusting the PC to ensure excitation of the fundamental TE mode in the microdisk resonator. The pump electric field is aligned parallel to the *b*-axis of the NbOI_2_ crystal (marked with a red arrow). According to Figure 1f, this configuration yields the maximum second-harmonic signal.

Laser frequency scanning was performed in the 1,540–1,560 nm wavelength range. The output raw data from the oscilloscope is shown in Figure S10. The laser (Aunion Tech Co., Ltd) scanned at a speed of 1 nm/s. To obtain the second harmonic signals, we tuned the pump wavelength to the resonant wavelength of the microcavity (1,540.6 nm and 1,551.9 nm). Figure 3b shows the transmission spectrum at the resonant wavelength of 1,540.6 nm, with a Lorentzian-fitted linewidth of ∼0.2 nm; Figure 3c displays the SHG spectra under varying pump powers (175 μW–350 μW, the specific values for the pump power are as follows: 175 μW, 187 μW, 201 μW, 214 μW, 231 μW, 250 μW, 270 μW, 291 μW, 308 μW, 336 μW and 350 μW), where the second-harmonic wavelength of 770.3 nm corresponds to half of the pump wavelength; Additionally, the quadratic dependence of the SHG intensity on pump power in a log scale (fitted slope: 2.01 ± 0.03) further confirms the second-order nonlinear optical nature of the SHG process (Figure 3d). We also investigated the transmission spectrum at a resonant wavelength of 1,551.9 nm (Figure 3e), the corresponding SHG spectra under varying pump powers (Figure 3f) and the dependence of the SHG intensity on pump power in a log scale (Figure 3g). In Figure 3f, the pump power gradually increases from 184 μW to 410 μW, in the following order: 184 μW, 210 μW, 229 μW, 238 μW, 253 μW, 265 μW, 287 μW, 317 μW, 352 μW, 373 μW and 410 μW. Due to the resonance enhancement effect of the cavity, SHG process is achieved under the pump of a low-power CW laser.

Under experimental conditions identical to those used for Figure 3, we characterized a bare Si_3_N_4_ microdisk resonator. We sequentially tuned the pump wavelength to each resonant wavelength and performed SHG measurements. Although theoretically, cavity enhancement could amplify interface effects and contribute to measurable SHG [12], under our experimental conditions – specifically, a *Q*-factor on the order of 10^4^ and CW laser pumping at sub-milliwatt power levels – we did not detect any second-harmonic signal in the bare Si_3_N_4_ microdisk resonator. In contrast, the SHG process was only achieved after integrating the NbOI_2_ flake onto the Si_3_N_4_ microdisk resonator, which directly confirms that the collected SHG signal originates from NbOI_2_.

Next, we estimate the conversion efficiency of SHG: under the condition where the pump wavelength is 1,540.6 nm, the output power of the tapered fiber is 350 μW, which is used as the pump power *P*_pump_; For the generated second harmonic signal, considering the absorption loss of single-mode fiber in the communication band for visible light, the diffraction efficiency of the spectrograph, and the quantum efficiency, the estimated power for the generated second harmonic signal *P*_SHG_ is 29 pW. Therefore, the estimated conversion efficiency of SHG is: $\eta_{\text{integrated}}=\frac{P_{\text{SHG}}}{P_{\text{pump}}^{2}}$ × 100 % ≈ 0.024 %/W.

Although the conversion efficiency is relatively low, this process is indeed enabled by the cavity field enhancement. The high-*Q* microresonator (loaded *Q* ∼ 7,700) builds up the intracavity pump power significantly, making the SHG process feasible. This is a key advantage of our cavity-integrated approach, allowing us to study SHG process in NbOI_2_ without using pulsed lasers.

The spectral data in Figure 1g were obtained using a 100× objective with a collection efficiency of approximately 12 % for the second-harmonic signal at 532 nm. Based on this, the SHG conversion efficiency of pure NbOI_2_ under free-space configuration at 400 μW pump power was calculated to be approximately *η*_space,1064_ = 2.04 × 10^−6^ %/W. After wavelength scaling to 1,550 nm, we obtain an cavity-enhancement factor of approximately *η*_integrated_/*η*_space,1550_ ≈ 25,000.

Table 1 summarizes the progress made in integrating 2D materials with high intrinsic *χ* ^(2)^ with WGM microcavities for SHG research. In all cases, the pump sources are continuous-wave lasers operating in the communication band; ${\chi_{\text{intrinsic}}}^{\left(2\right)}$ represents the second-order nonlinear polarizability of the 2D material; and the microcavity platforms used include Si_3_N_4_ microring resonators (MRR), SiO_2_ microsphere resonators (MSR), and Si_3_N_4_ microdisk resonators (MDR) used in this work. “*Q* factor (before)” and “*Q* factor (after)” presents the *Q* factors for the microcavities before and after the integration with 2D materials. The Si_3_N_4_ microdisk resonator platform used in our work offers distinct advantages over other resonator architectures: Compared to microring resonators, its fiber coupling configuration allows more flexible and robust critical coupling control under fabrication variations; relative to microspheres, our fully planar platform provides superior integration compatibility with 2D materials and complementary metal-oxide-semiconductor (CMOS) compatibility while maintaining high *Q*-factors. Furthermore, unlike Fabry–Perot cavities that offer high *Q* but lack mode discreteness [31], or nanoplasmonic systems that provide strong spatial confinement but suffer from broad resonances and high loss [32], the microdisk resonator platform employed in this work simultaneously combines high-*Q* temporal confinement, a discrete mode spectrum for phase matching, and a flexible and robust experimental testing configuration, making it an ideal and highly promising platform for nonlinear enhancement studies of 2D materials. Although a direct comparison of effective nonlinear coefficients ( ${\chi_{\text{eff}}}^{\left(2\right)}$ ) across different 2D materials-integrated microcavity platforms is infeasible due to the lack of reported values, the excellent second-order nonlinear optical properties of NbOX_2_ confirmed by free-space optical studies [22], [23], combined with the high SHG enhancement factor achieved in our microcavity platform, strongly demonstrate that the NbOX_2_-integrated Si_3_N_4_ microdisk resonator proposed in this work possesses highly competitive effective second-order nonlinearity and significant potential for further optimization.

**Table 1:** Summary of SHG research by integrating 2D materials with WGM microcavity platforms.

Materials	${\chi_{\mathrm{intrinsic}}}^{\left(2\right)}$ (pm/V)	Microcavities	*Q* factor (before)	*Q* factor (after)	*η* (%/W)	Ref.
GaSe	2,400	Si_3_N_4_ MRR	2,000	1,800	849	[33]
SnP_2_Se_6_	1,300	Si_3_N_4_ MRR	–	1,000	43.2	[34]
NbOBr_2_	91.6	Si_3_N_4_ MRR	14,000	24,000	158	[35]
WSe_2_	∼10^3^	SiO_2_ MSR	1 × 10^8^	5 × 10^6^	6.6 × 10^−4^	[36]
Surface molecule	210	SiO_2_ MSR	6 × 10^7^	3 × 10^7^	6.7	[37]
WS_2_	∼10^3^	SiO_2_ MSR	–	∼10^6^	1.08 × 10^−5^	[38]
NbOI_2_	∼90	Si_3_N_4_ MDR	29,200	7,700	0.024	This work

We can analyze the reasons for the low SHG conversion efficiency of the device in this work from a qualitative perspective: (1) Relatively low *Q* value of microcavity (for the bare Si_3_N_4_ microdisk, *Q* ∼ 10^4^), which can be improved by subsequent ultra-low-loss microcavity design; (2) In the phase-matching analysis in Figure 2g, the second-harmonic mode corresponding to the phase-matching point is the asymmetric TM_3_ mode, resulting in a low mode overlap between the fundamental mode and the second-harmonic mode. This can be optimized by the design of the geometric parameters of the microcavity and gradient thickness engineering of NbOI_2_; (3) The absorption of the generated second harmonic signal by the NbOI_2_ material itself (Figure 1a) is also a factor contributing to the relatively low calculated *η*_integrated_; (4) For the pump of light and the extraction of the second harmonic signal, we used the same tapered fiber, which has absorption for the second harmonic signal, also leading to a lower calculated *η*_integrated_. The experimental setup can be optimized in subsequent work by employing two separate tapered fibers for the coupling of the pump and the extraction of the second-harmonic signal, respectively [12]. Optimizing these limiting factors will further improve the SHG conversion efficiency.

We have also theoretically analyzed the maximum achievable SHG conversion efficiency (Supporting Material Note S2), projecting a potential improvement of two orders of magnitude over the current highest reported SHG conversion efficiency listed in Table 1. Beyond cavity-induced SHG enhancement, the intrinsic *χ* ^(2)^ of NbOI_2_ could be further enhanced via band-edge resonance when the laser energy approaches half the bandgap of the NbOI_2_: for NbOI_2_ with a bandgap of ∼1.74 eV, this implies that tuning the microdisk resonance to approximately 1,427 nm (corresponding to a photon energy of ∼0.87 eV) could theoretically maximize its intrinsic *χ* ^(2)^ response. This would enable dual-resonance enhancement, combining pump photon energy resonance with optical microcavity resonance, thereby pushing the overall second-order nonlinear response of the system to a new level, further demonstrating the significant application potential of our proposed NbOI_2_-integrated Si_3_N_4_ microdisk resonator platform in the field of integrated second-order nonlinear optics.

## 3 Conclusions

In this study, NbOI_2_-integrated Si_3_N_4_ microdisk resonators were successfully fabricated through a vdW integration strategy, and SHG was realized under the pump of a low-power (∼175 μW) CW laser and the SHG conversion efficiency was calculated to be 0.024 %/W. By leveraging the exceptional second-order nonlinear optical properties of 2D NbOI_2_ with the strong optical confinement capability of the Si_3_N_4_ microdisk, we overcame the optical path length limitation imposed by the nanoscale thickness of 2D materials in traditional free-space optical systems. Furthermore, the site-specific vdW transfer technique, combined with NbOI_2_’s intrinsic lack of inversion symmetry (independent of layer parity), greatly shortens the time required for device preparation. In future experiments, polarization-resolved SHG measurements should be conducted to fully characterize the anisotropic nonlinear response within the cavity; meanwhile, electro-optic tunable frequency conversion devices can be developed by leveraging the ferroelectric properties of NbOX_2_ materials. This work not only establishes a universal strategy for the co-design of 2D materials and microcavities but also opens new avenues for integrated nonlinear photonics in quantum technologies and metrological systems.

## Supplementary Material

Supplementary Material Details
