# Supplementary material for: Second-harmonic generation in NbOI2-integrated silicon nitride microdisk resonators
Source: Nanophotonics. 2025 Nov 7;14(27):5337–45. doi: 10.1515/nanoph-2025-0428 (PMC12717909; doi:10.1515/nanoph-2025-0428)
Supplement: Supplementary file 1 — Supplementary Material Details [file j_nanoph-2025-0428_suppl_001.docx]

Supporting Material for

**Second-harmonic generation in NbOI_2_-integrated silicon nitride
microdisk resonators**

Ning Liu^1,2^, Qiang Liu^1,2^, Yutian Lin^1,2^, Zhihong Zhu ^1,2,*^, and Ken Liu^1,2,*^

^1^College of Advanced Interdisciplinary Studies & Hunan Provincial Key Laboratory of Novel Nano-Optoelectronic Information Materials and Devices, National University of Defense Technology, Changsha 410073, China.

^2^ Nanhu Laser Laboratory, National University of Defense Technology, Changsha 410073, China.

*Corresponding authors: Zhihong Zhu, [zzhwcx@163.com](mailto:zzhwcx@163.com), Ken Liu, [liukener@163.com](mailto:liukener@163.com)

**Table of Content**

**Note S1:** Robustness and versatility of phase matching technology by designing geometry

**Note S2:** Analysis of SHG conversion efficiency

**Figure S1:** Acquisition of 2D NbOI_2_

**Figure S2:** Optical absorption spectrum of 2D NbOI_2_ and corresponding $\left( \alpha h\nu\right)^{1/2}-h\nu$ plot

**Figure S3:** Stability of 2D NbOI_2_ under ambient conditions

**Figure S4:** Schematic illustrations of the atomic structure of NbOI_2_

**Figure S5:** Fabrication process flow of the suspended Si_3_N_4_ microdisks

**Figure S6:** Wet etching rate of SiO_2_

**Figure S7:** OM image of a typical Si_3_N_4_ microdisk used in the experiment

**Figure S8:** OM images of the 2D NbOI_2_ and the PVA/NbOI_2_/Si_3_N_4_ microdisk heterostructure

**Figure S9:** Phase matching analysis of a Si_3_N_4_ microdisk resonator integrated with 30nm-thick NbOI_2_ flake.

**Figure S10:** Raw transmission spectrum of the Si_3_N_4_ microdisk integrated with NbOI_2_ at 1540-1560 nm.

**
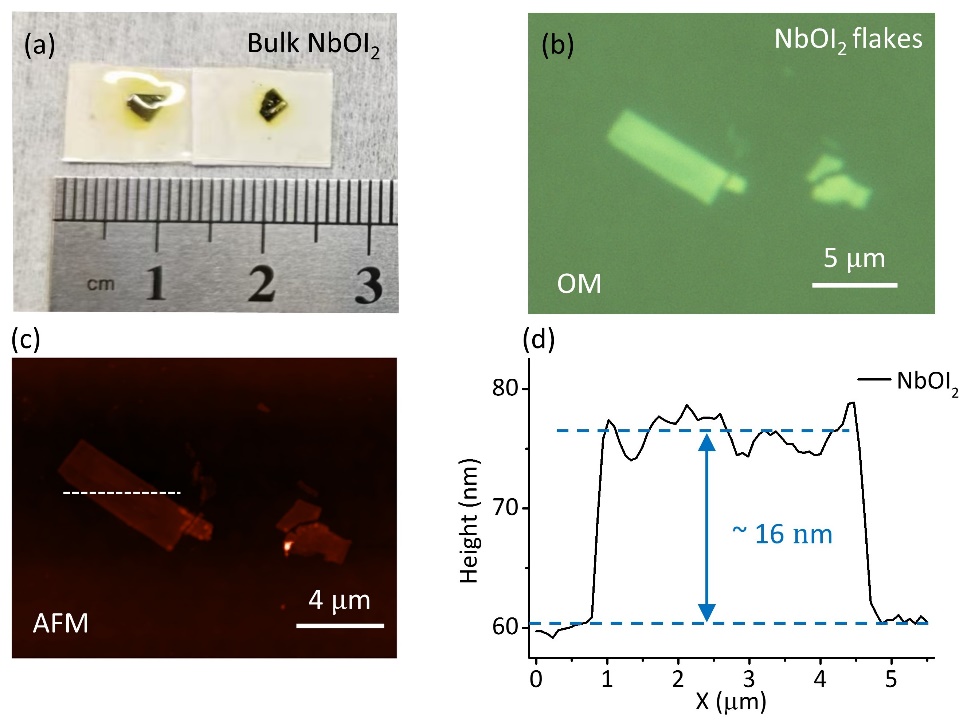
**

**Figure S1.** Acquisition of 2D NbOI_2_. (a) Optical image of bulk NbOI_2_ crystals. (b) Optical microscopy (OM) image of 2D NbOI_2_ flakes obtained via mechanical exfoliation. (c) Atomic force microscopy (AFM) image corresponding to the NbOI_2_ flakes in (b). (d) Height profile along the white dashed line in (c), indicating a thickness of ~16 nm for the NbOI_2_ flake.


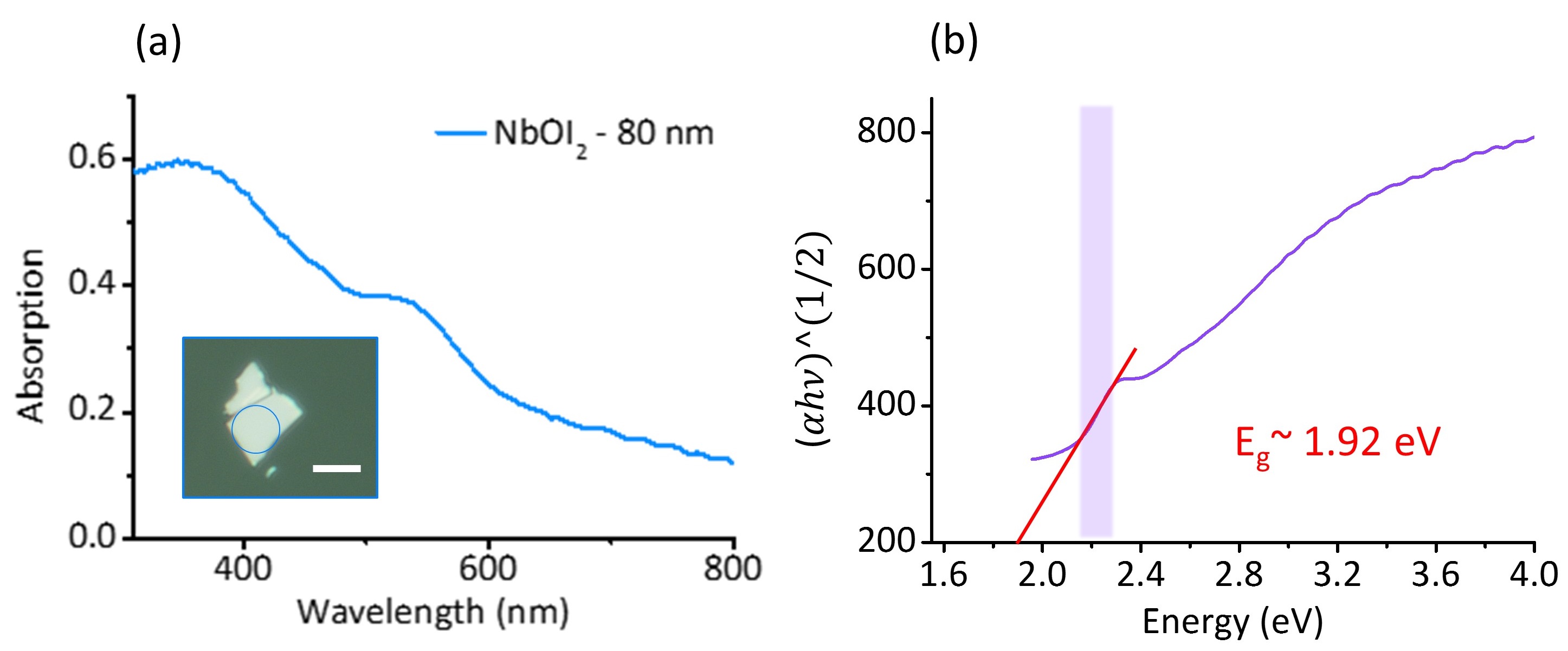


**Figure S2.** Optical absorption spectrum of 2D NbOI_2_ and corresponding $\left( \alpha h\nu\right)^{1/2}-h\nu$ plot. (a) Optical absorption spectrum of a 80 nm-thick NbOI_2_ flake. Inset: OM image of the sample for absorption measurement, with the blue circle marking the tested region. Scale bar: 5 μm. (b) $\left( \alpha h\nu\right)^{1/2}-h\nu$ plot derived from the absorption spectrum in (a) (purple shaded area denotes the linear fitting region), yielding a bandgap of ~1.92 eV for the 90 nm-thick NbOI_2_ flake.

**
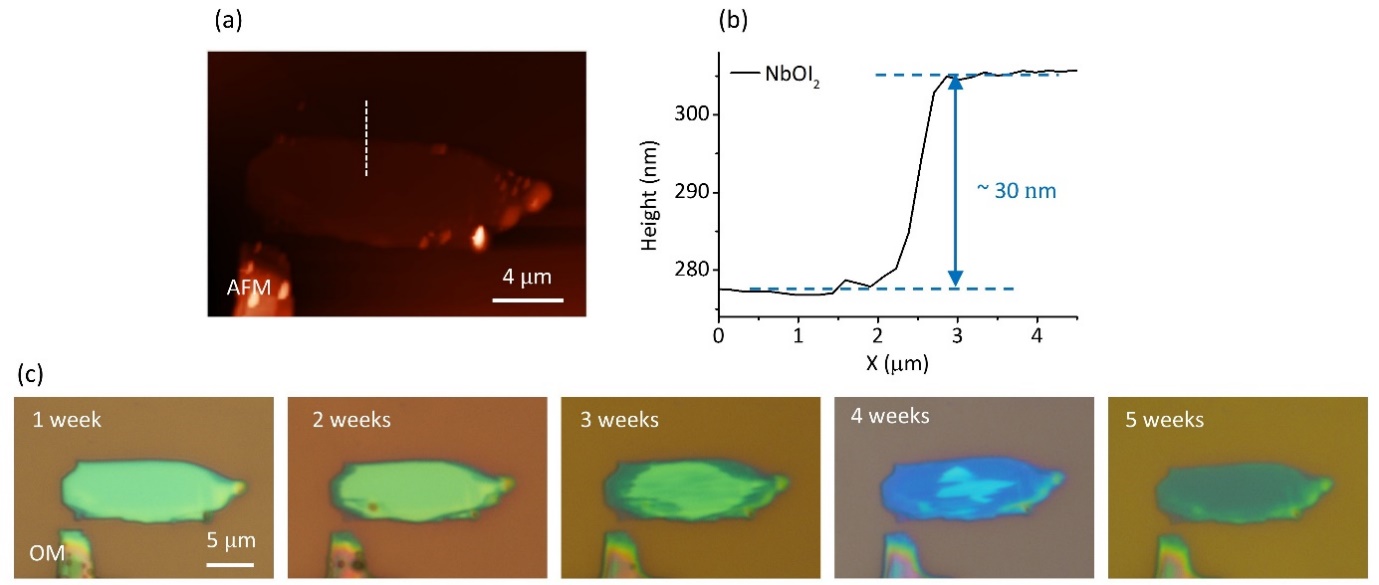
**

**Figure S3.** Stability of 2D NbOI_2_ under ambient conditions. (a) AFM image of the 2D NbOI_2_ in Figure 1c. (b) Height profile along the white dashed line in (a), indicating a thickness of ~30 nm. (c) OM images of time-dependent degradation of the NbOI_2_ flake under ambient conditions (~60% relative humidity).

**
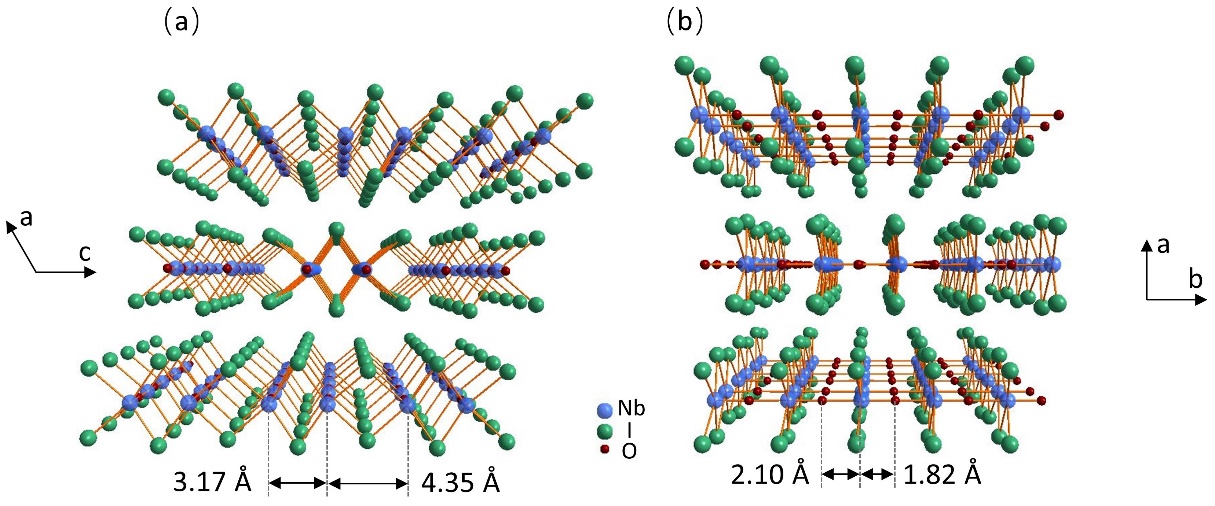
**

**Figure S4.** Schematic illustrations of the atomic structure of NbOI_2_. (a) Side view of the NbOI₂ structure along the *c*-axis, showing an alternation in Nb–Nb distances. (b) Side view along the *b*-axis, highlighting alternating variations in Nb–O bond lengths.

**
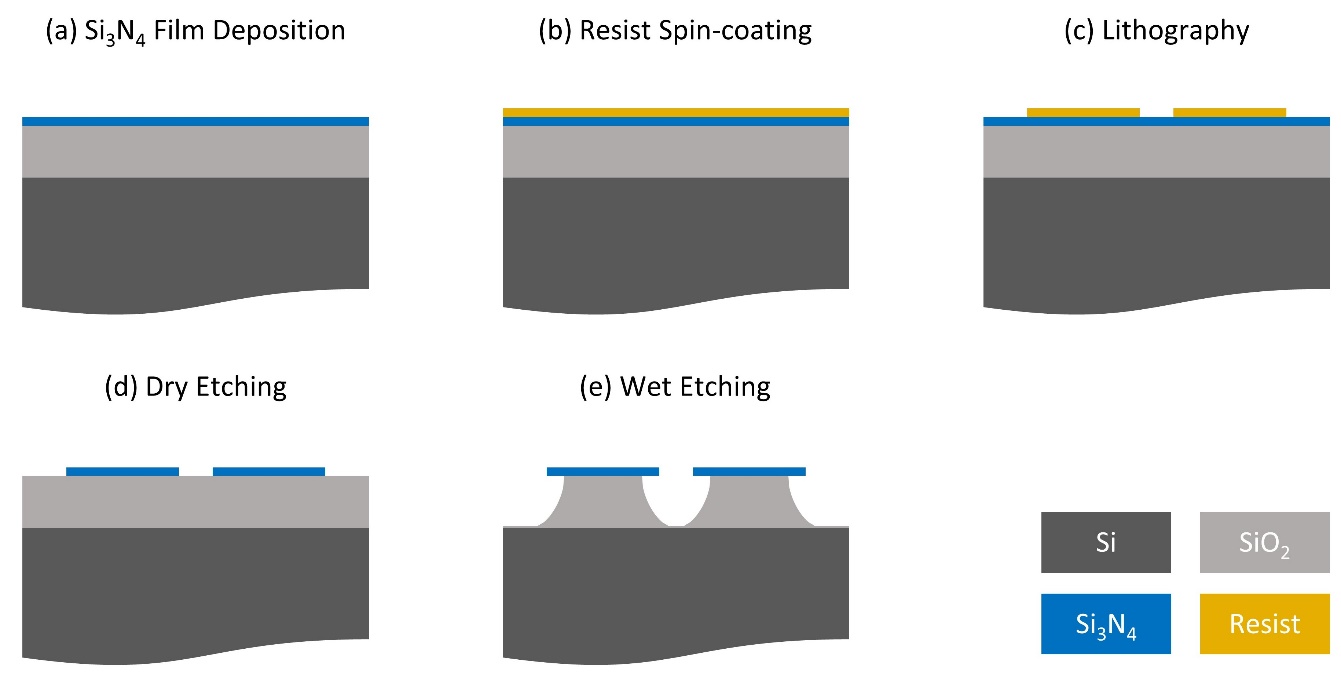
**

**Figure S5.** Fabrication process flow of the suspended Si_3_N_4_ microdisks. (a) Deposition of a Si_3_N_4_ thin film on a SiO_2_/Si substrate. (b) Spin-coating of photoresist. (c) Lithography for microdisk patterning. (d) Dry etching to transfer the pattern to the Si_3_N_4_ layer. (e) Wet etching of SiO_2_ to release the Si_3_N_4_ microdisks.

**
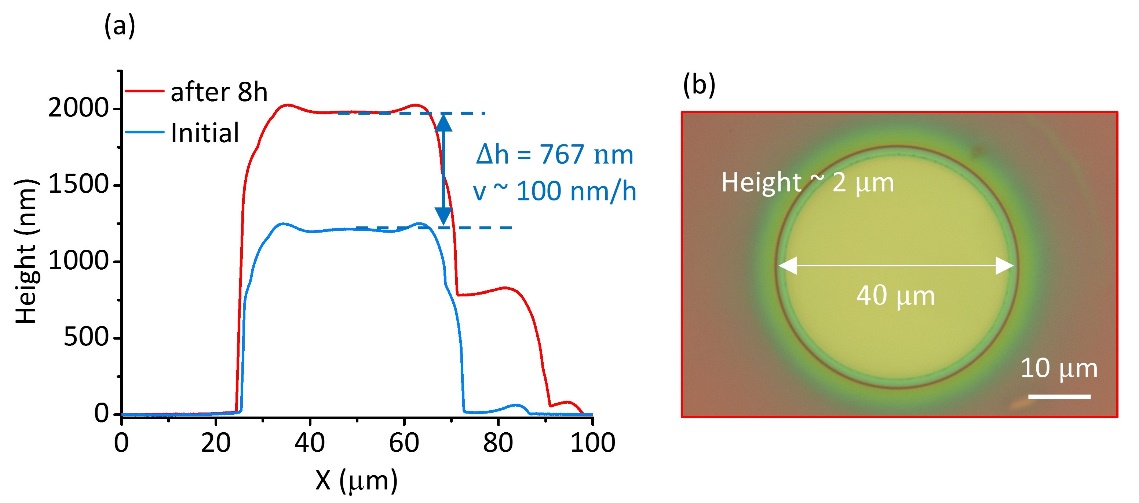
**

**Figure S6.** Wet etching rate of SiO_2_. (a) Test results of surface-to-substrate height on the Si_3_N_4_ microdisk before (blue line) and after (red line) 8 hours of wet etching, demonstrating a SiO₂ etching rate of ~100 nm/h. The etching was performed with 1/3 mol/L KOH solution under an 80°C water bath. (b) OM image of the Si_3_N_4_ microdisk. The height difference between the upper surface of Si_3_N_4_ microdisk and the substrate is about 2 μm, and the diameter of the microdisk is 40 μm.

**
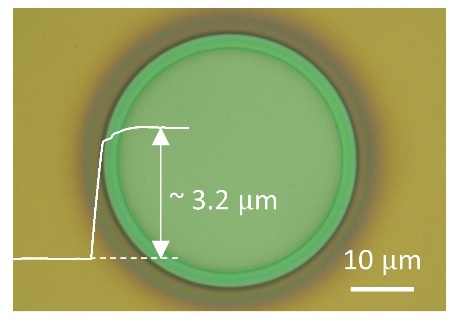
**

**Figure S7.** OM image of a typical Si_3_N_4_ microdisk used in the experiment. Inset: The white line indicates the height difference (~3.2 μm) between the top surface of the SiN microdisk and the substrate.

**
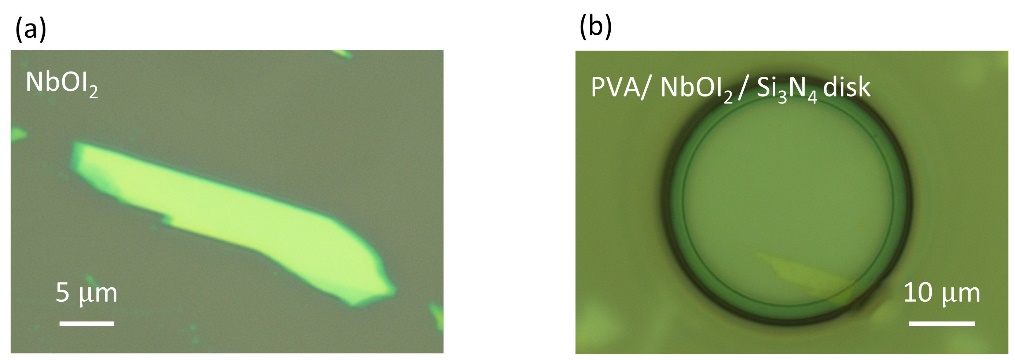
**

**Figure S8.** (a) OM image for the 2D NbOI_2_ in Figure 2c. (b) OM image of the PVA/NbOI_2_/ Si_3_N_4_ microdisk heterostructure.


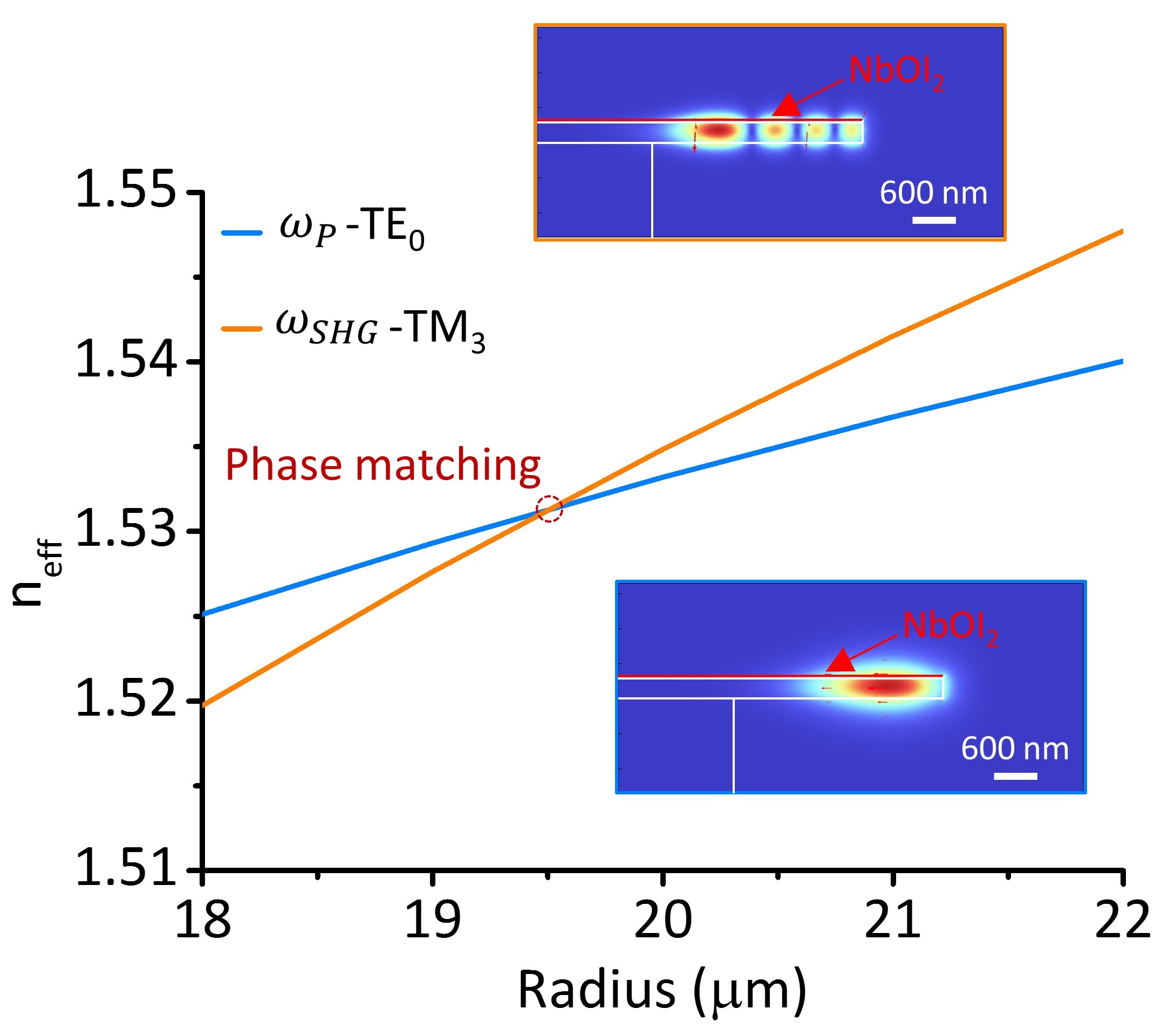


**Figure S9.** Effective refractive index (n_eff_) curves of the TE_0_ mode at the fundamental frequency ($\omega_{P}$-TE_0_, blue line) and the TM_3_ mode at the second harmonic frequency ($\omega_{SHG}$-TM_3_, orange line) as functions of the microdisk diameter for a Si_3_N_4_ microdisk integrated with a 30 nm-thick NbOI_2_ flake. The intersection at a radius of ~19.6 μm indicates the phase-matching point. Insets: Electric field distributions of the TE_0_ (fundamental frequency) and TM_3_ (second-harmonic frequency) modes.


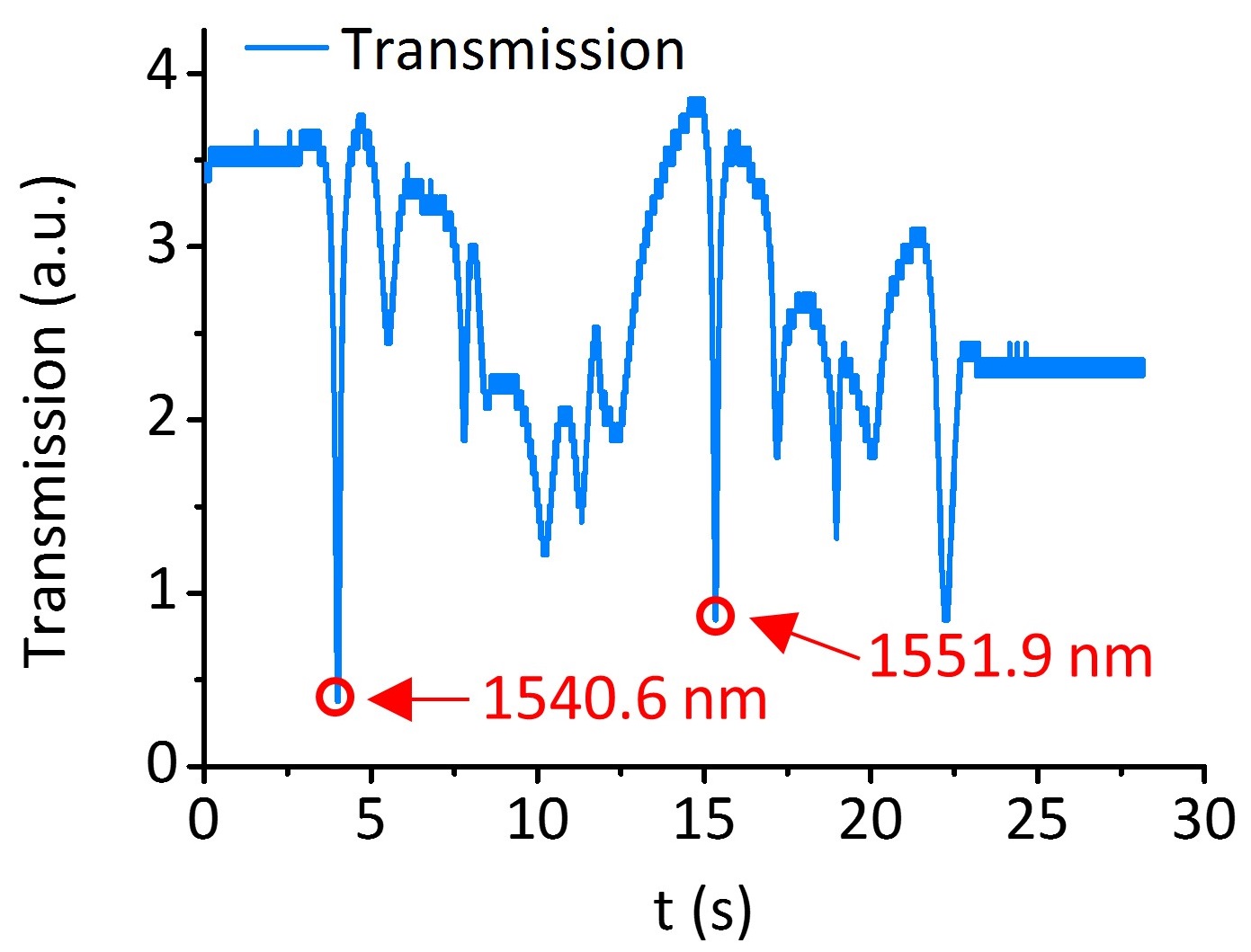


**Figure S10.** Raw transmission spectrum of the Si_3_N_4_ microdisk integrated with NbOI_2_ at 1540-1560 nm, where the two resonant wavelengths studied in Figure 3 are marked with red circles.

**Note S1:** Robustness and versatility of phase matching technology by designing geometry

The phase-matching achieved through photonic design is indeed highly versatile and serves as a powerful tool for engineering a wide range of nonlinear processes.

(1) Different nonlinear spectroscopic techniques (e.g., pump-probe, double quantum coherence) require specific phase-matching conditions. In bulk optics, this is controlled by the alignment of the wavevectors of the free-space beams. In our integrated microcavity platform, an analogous, but far more precise, control is achieved by designing the geometry (e.g., radius, waveguide width, and etch depth) to tailor the dispersion of the optical modes. This allows us to target the specific resonance conditions required for a desired nonlinear process: for sum-frequency generation (SFG), the cavity can be designed to support three resonant modes satisfying $\omega_{3}=\omega_{1}{+\omega}_{2}$ and m_3_ = m_1_+m_2_; for difference-frequency generation (DFG), the conditions would be $\omega_{3}=\omega_{1}{-\omega}_{2}$ and m_3_ = m_1_ - m_2_; for pump-probe experiments, the same principle applies. The cavity can be engineered to resonate at both the pump and probe wavelengths, dramatically enhancing the interaction strength for sensitive measurements like cross-phase modulation or two-photon absorption.

Therefore, the photonic design is not limited to SHG; it is a general-purpose methodology for achieving phase-matching in a vast landscape of $\chi^{(2)}$and $\chi^{(3)}$ nonlinear optics.

(2) Robustness and Post-Fabrication Tuning. Once fabricated, the phase-matching condition is inherently stable and locked into the device geometry, immune to mechanical vibrations or misalignment, which is a significant advantage over free-space setups. While the fundamental phase-matching is set by geometry, several post-fabrication tuning techniques are well-established to compensate for manufacturing tolerances or to actively switch between processes, such as “thermal tuning”, “carrier-effect tuning”, “strain tuning” and so on. These methods allow for fine-tuning the phase-matching condition with high precision, making the platform both robust and adaptable.

**Note S2:** Analysis of SHG conversion efficiency

We have employed nonlinear coupled-mode theory to estimate the maximum achievable SHG efficiency in the NbOI_2_-integrated Si_3_N_4_ microdisk system.

The SHG process in a microdisk resonator involves two optical modes: mode *a* at the fundamental frequency (with angular frequency *ω*) and mode *b* at the second-harmonic frequency (with angular frequency *ω_b_* ≈ 2*ω_a_*). When the angular frequency of the pump light *ω_p_* approaches *ω_a_*, the microdisk system can be described by the following Hamiltonian [S1]:

|  | $H=\omega_{a}a^{*}a+\omega_{b}b^{*}b+g\left[ \left( a^{*} \right)^{2}b+a^{2}b^{*} \right]+\varepsilon_{p}\left( ae^{i\omega_{p}t}+a^{*}e^{-i\omega_{p}t} \right.)$, | (1) |
| --- | --- | --- |

Where, *a* and *b* represent the Bosonic operators for the fundamental mode and second-harmonic mode, respectively; $\varepsilon_{p}=\sqrt{\frac{2\kappa_{a,1}P_{p}}{\hbar\omega_{p}}}$ represents the pump field strength, $P_{p}$ is the pump power, $\kappa_{a,1}$ is the external coupling rate between mode *a* and the coupling waveguide, $g$ is the nonlinear coupling strength between mode *a* and mode *b*:

|  | $\hbar g=\varepsilon_{0}\iiint d\theta dr dz \frac{3\chi^{\left( 2 \right)}\left( r \right)}{4\sqrt{2}}\left[ u_{a,z}^{*}\left( r \right) \right]^{2}u_{b,z}\left( r \right)$, | (2) |
| --- | --- | --- |

where $\varepsilon_{0}$ is the vacuum permittivity, $\chi^{\left( 2 \right)}\left( r \right)$ denotes the second-order susceptibility of the medium, $u_{a\left( b \right),z}\left( r \right)$ represents the electric field profiles of mode *a*(*b*) within the microdisk resonator, expressed in polar coordinates.

Next, we introduce the effective mode overlap factor:

|  | $\zeta=\frac{\iint dr dz \left[ u_{a,z}^{*}\left( r,z \right) \right]^{2}u_{b,z}\left( r,z \right)}{\iint dr dz \left\vert u_{a,z}\left( r,z \right) \right\vert^{2}\sqrt{\iint dr dz \left\vert u_{b,z}\left( r,z \right) \right\vert^{2}}}$. | (3) |
| --- | --- | --- |

So

|  | $g\approx\zeta\sqrt{\frac{\hbar\omega_{a}^{2}\omega_{b}}{\varepsilon_{0}2\pi R}\cdot\frac{1}{\varepsilon_{a}\sqrt{\varepsilon_{b}}}\cdot\frac{3\chi^{\left( 2 \right)}}{4\sqrt{2}}}\times\delta\left( m_{b}-2m_{a} \right)$, | (4) |
| --- | --- | --- |

where $\varepsilon_{a(b)}$ represents the relative permittivity of mode *a*(*b*), which depends on the angular frequency and spatial position; *m_a_*(*m_b_*) denotes the angular momentum of mode *a*(*b*). For the Kronecker delta function *δ*(*m_b_* - 2*m_a_*), the coupling strength $g$ assumes a non-zero value only when momentum conservation condition *m_b_* - 2*m_a_* = 0 is satisfied.

Under CW pumping conditions, using the non-depletion approximation, the SHG conversion efficiency can be expressed in the following form:

|  | $\eta=\frac{P_{\text{SHG}}}{P_{p}^{2}}=g^{2}\frac{2\kappa_{b,1}}{\delta_{b}^{2}+\kappa_{b}^{2}}\left( \frac{2\kappa_{a,1}}{\delta_{a}^{2}+\kappa_{a}^{2}} \right)^{2}\frac{\hbar\omega_{b}}{\left( \hbar\omega_{p} \right)^{2}}.$ | (5) |
| --- | --- | --- |

where *δ_a_* = *ω_a_* - *ω_p_* (*δ_b_* = *ω_b_* - 2*ω_p_*) represents the angular frequency detuning of mode *a*(*b*) relative to the pump; *κ_a_* = *κ_a_*_,1_ + *κ_a_*_,0_ (*κ_b_* = *κ_b_*_,1_ + *κ_b_*_,0_) denotes the total loss rate of cavity mode *a*(*b*), specifically including the external loss rate *κ_a_*_(_*_b_*_),1_ and the intrinsic loss rate *κ_a_*_(_*_b_*_),0_. When the pump frequency is tuned to match the frequency of cavity mode *a*, *δ_a_* = *ω_a_* - *ω_p_* = 0; when the energy conservation condition is satisfied in the SHG process, i.e., *ω_b_* ≈ 2*ω_a_*, *δ_b_* = *ω_b_* - 2*ω_p_* = 0 is automatically satisfied, indicating that the double-resonance condition is met in the second-harmonic conversion process.

At this point,$\eta$ can be further simplified to:

|  | $\eta=\frac{16g^{2}}{\left( \frac{\kappa_{b,0}}{\sqrt{\kappa_{b,1}}}+\sqrt{\kappa_{b,1}} \right)^{2}\left( \frac{\kappa_{a,0}}{\sqrt{\kappa_{a,1}}}+\sqrt{\kappa_{a,1}} \right)^{4}}\cdot\frac{1}{\hbar\omega_{p}}$. | (6) |
| --- | --- | --- |

Furthermore, when both modes satisfy the critical coupling condition, i.e., *κ_a_*_,1_ = *κ_a_*_,0_，*κ_b_*_,1_ = *κ_b_*_,0_, the maximum SHG conversion efficiency can be expressed as:

|  | $\eta_{max}=\frac{g^{2}}{4\kappa_{b,0}\kappa_{a,0}^{2}}\cdot\frac{1}{\hbar\omega_{p}}=\frac{g^{2}Q_{a,0}^{2}Q_{b,0}}{\hbar\omega_{a}^{4}}$. | (7) |
| --- | --- | --- |

where $Q_{a,0}=\frac{\omega_{a}}{2\kappa_{a,0}}$，$Q_{b,0}=\frac{\omega_{b}}{2\kappa_{b,0}}$ represent the intrinsic Q factors of modes *a* and *b*.

Under the phase matching analysis of the microdisk shown in Figure 2(f), energy and momentum conservation are satisfied. Assuming the nonlinear coupling strength $g$ remains constant, subsequent experimental optimizations can increase the intrinsic quality factor of the microcavity by two orders of magnitude (the intrinsic Q of the bare Si_3_N_4_ microcavity rises from ~10⁴ to ~10⁶, with corresponding estimated $Q_{a,0}$ and $Q_{b,0}$ also improving by two orders of magnitude). Under critical coupling conditions, the SHG conversion efficiency can be improved to 24,000 %/W. This value exceeds the highest reported in comparable works (as listed in Table 1) by two orders of magnitude, demonstrating the significant application potential of our proposed NbOI_2_-integrated Si_3_N_4_ microdisk resonator in the field of integrated second-order nonlinear optics.

**Reference**

[S1] X. Guo, C.-L. Zou, and H. X. Tang, "Second-harmonic generation in aluminum nitride microrings with 2500%/W conversion efficiency," *Optica*, vol. 3, no. 10, pp. 1126-1131, 2016.
